# Deletion of Genes Encoding Arginase Improves Use of “Heavy” Isotope-Labeled Arginine for Mass Spectrometry in Fission Yeast

**DOI:** 10.1371/journal.pone.0129548

**Published:** 2015-06-15

**Authors:** Weronika E. Borek, Juan Zou, Juri Rappsilber, Kenneth E. Sawin

**Affiliations:** 1 Wellcome Trust Centre for Cell Biology, School of Biological Sciences, University of Edinburgh, Edinburgh, United Kingdom; 2 Department of Bioanalytics, Institute of Biotechnology, Technische Universität Berlin, Berlin, Germany; St Jude Children's Research Hospital, UNITED STATES

## Abstract

The use of “heavy” isotope-labeled arginine for stable isotope labeling by amino acids in cell culture (SILAC) mass spectrometry in the fission yeast *Schizosaccharomyces pombe* is hindered by the fact that under normal conditions, arginine is extensively catabolized *in vivo*, resulting in the appearance of “heavy”-isotope label in several other amino acids, most notably proline, but also glutamate, glutamine and lysine. This “arginine conversion problem” significantly impairs quantification of mass spectra. Previously, we developed a method to prevent arginine conversion in fission yeast SILAC, based on deletion of genes involved in arginine catabolism. Here we show that although this method is indeed successful when ^13^C_6_-arginine (Arg-6) is used for labeling, it is less successful when ^13^C_6_^15^N_4_-arginine (Arg-10), a theoretically preferable label, is used. In particular, we find that with this method, “heavy”-isotope label derived from Arg-10 is observed in amino acids other than arginine, indicating metabolic conversion of Arg-10. Arg-10 conversion, which severely complicates both MS and MS/MS analysis, is further confirmed by the presence of ^13^C_5_^15^N_2_-arginine (Arg-7) in arginine-containing peptides from Arg-10-labeled cells. We describe how all of the problems associated with the use of Arg-10 can be overcome by a simple modification of our original method. We show that simultaneous deletion of the fission yeast arginase genes *car1+* and *aru1+* prevents virtually all of the arginine conversion that would otherwise result from the use of Arg-10. This solution should enable a wider use of heavy isotope-labeled amino acids in fission yeast SILAC.

## Introduction

Stable isotope labeling by amino acids in cell culture (SILAC; [1]) combined with high-resolution mass spectrometry (MS) facilitates accurate and reliable relative quantification of large numbers of proteins from two or more samples and thus has become an important technique for quantitative proteomics. In SILAC, proteins are metabolically labeled using isotopically-labeled amino acids, typically arginine and lysine. Samples grown in “light” and “heavy” media (i.e. containing naturally-occurring or isotopically-labeled amino acids, respectively) are mixed together and analysed simultaneously by MS, and pairs of chemically identical peptides originating from the “light” and “heavy” cultures can be distinguished in the mass spectrometer on the basis of their mass difference. For such pairs of peptides (often referred to as peptide “isotopologues”, i.e. peptides that have the same sequence and differ only in their isotopic composition), MS intensity ratios are used to determine peptide and/or protein relative abundance. However, as a result of metabolic conversion of arginine to proline and other amino acids, SILAC studies can be compromised by errors in peptide quantification and/or peptide identification [2–6]. For example, for a given peptide, incorporation of “heavy” label into amino acids other than arginine will lead to the appearance of additional, higher-molecular-mass peaks in the mass spectrum of the peptide, producing an isotopic cluster that may differ considerably from the expected conventional isotopic envelope or, indeed, appear as two independent envelopes. Such additional peaks may not contribute to quantification of the labeled peptide, thus leading to underestimation of relative abundance of that peptide. Moreover, the “broadening” of isotopic clusters that results from additional peaks may lead to overlap of these clusters with the isotopic envelopes of neighboring peptides possessing similar mass-to-charge (m/z) ratios, further complicating analysis. In addition, as a consequence of arginine conversion, an MS peak chosen for fragmentation and MS/MS analysis may actually be a superposition of two different isotopic forms of the same peptide, which could negatively affect peptide identification. Finally, it is possible that, as a result of arginine conversion, different isotopologues of the same peptide may be selected for fragmentation multiple times, decreasing the overall sensitivity of global MS analysis.

Fission yeast *Schizosaccharomyces pombe* is easy to grow, highly amenable to genetic manipulations, and an excellent model organism for the investigation of a broad range of eukaryotic cellular processes [7–12] many of which can be studied on a proteome-wide scale [2, 13–15]. Given the growing importance of quantitative proteomics, generation of generic tools allowing for efficient SILAC application in fission yeast is of great importance.

We previously reported a genetic engineering method for preventing conversion of ^13^C_6_-arginine (Arg-6) in fission yeast SILAC [2], and this general approach was later successfully applied to nematodes [3] and budding yeast [16]. In that previous work, we showed that prevention of Arg-6 conversion into glutamate, glutamine and proline can be achieved either by deletion of the single fission yeast ornithine transaminase gene, *car2+*, or by simultaneous deletion of the two arginase genes, *car1+* and *aru1+*. Here we show that when *car2∆* cells are labeled with ^13^C_6_^15^N_4_-arginine (Arg-10), heavy-isotope label is converted into ^13^C_6_^15^N_1_-arginine (Arg-7) as well as other metabolic products, leading to the appearance of highly complex isotopic clusters that would significantly hinder SILAC experiments. Detailed analysis of results from Arg-10 labeling of *car2∆* cells led us to hypothesize that the problems associated with Arg-10 labeling may be due to arginase activity in these cells. Accordingly, we confirm experimentally that these Arg-10-specific problems can indeed be overcome by using *car1∆ aru1∆* double mutant cells instead of car2∆ single mutants. This modified genetic engineering solution should allow more effective use of both Arg-6 and Arg-10 labels in fission yeast SILAC.

## Results and Discussion

Historically, many SILAC experiments have involved the use of Arg-6 and ^13^C_6_-lysine (Lys-6), because these were among the most readily-available heavy-isotope versions of arginine and lysine, and they are well-separated from unlabeled arginine and lysine [2, 4, 17, 18]. More recently, Arg-10 and ^13^C_6_^15^N_2_-lysine (Lys-8) have become widely available and indeed are now usually less expensive than Arg-6 and Lys-6. Use of Arg-10 and Lys-8 offers several potential advantages over Arg-6 and Lys-6, including: 1) better separation of “light” (Lys-0, Arg-0) vs. “heavy” spectra; 2) better identification of isotopically-labeled peptides, because of the different numbers of “heavy” atoms on arginine vs. lysine; and 3) the possibility for use in triple-label SILAC [19, 20]. With this in mind, we sought to expand the range of isotopically-labeled amino acids suitable for use in fission yeast SILAC experiments.

As part of a proteomics study of fission yeast microtubule nucleation protein Mto2 [21–23], we analyzed a GFP-tagged mutant form of Mto2, Mto2[17A]-GFP, expressed in *car2∆ arg1-230 lys3-37* auxotrophic cells grown in “light” unlabeled medium (Arg-0, Lys-0) or in two different types of “heavy” labeled medium (Arg-6, Lys-8; and Arg-10, Lys-8). LC-MS/MS analyses of equal amounts of anti-GFP immunoprecipitates derived from these three cultures yielded significantly different numbers of tryptic peptides identified in each sample (6677, 4609 and 1681 unique peptides detected with MASCOT scores >20 from (Arg-0, Lys-0), (Arg-6, Lys-8), and (Arg-10, Lys-8) cultures, respectively; data not shown).

**Fig 1:**
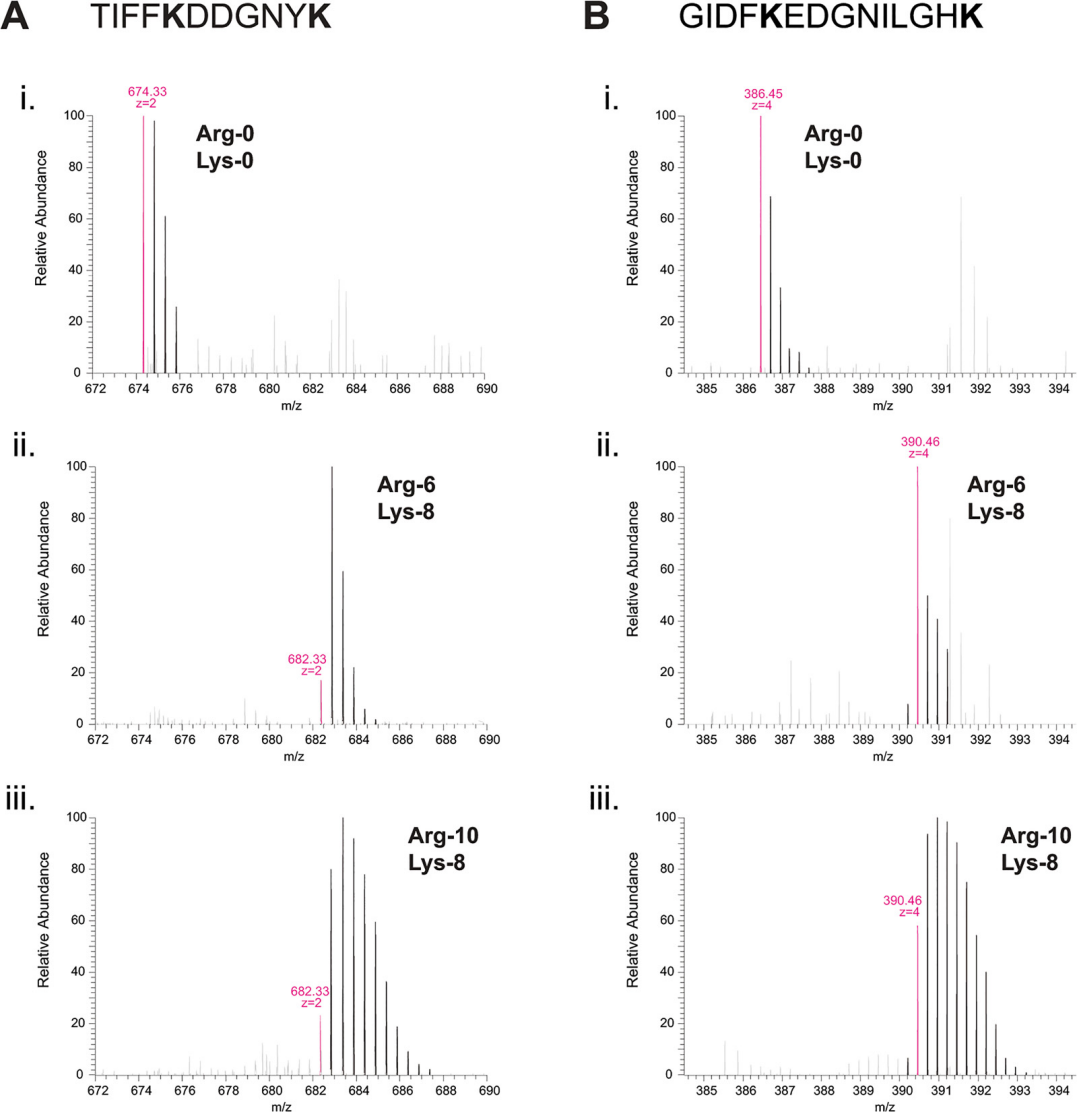
“Heavy”-isotope label from ^13^C_6_^15^N_4_-arginine (Arg-10) is converted into other amino acids in fission yeast. Mass spectra of tryptic peptides (TIFFKDDGNYK and GIDFKEDGNILGHK in (A) and (B), respectively) from *S*. *pombe* Mto2[17A]-GFP fusion protein isolated from *car2∆ arg1-230 lys3-37* cells grown in either (i) unlabeled arginine (Arg-0) and unlabeled lysine (Lys-0), (ii) ^13^C_6_-arginine (Arg-6) and ^13^C^15^N_2_-lysine (Lys-8), or (iii) ^13^C_6_^15^N_4_-arginine (Arg-10) and ^13^C_6_^15^N_2_-lysine (Lys-8), as indicated. In peptides from cells grown in Arg-10, additional higher-molecular-mass peaks are observed (iii), indicating conversion of “heavy”-isotope label into other amino acids. Such peaks are not observed from cells grown in Arg-6. To simplify comparison, peptides shown here do not contain arginine residues, so the masses of monoisotopic peaks of peptides from cells grown in Arg-6 and Arg-10 are identical (see Fig 2). Mass-to-charge (m/z) ratios of monoisotopic peaks and inferred peptide charge-states are indicated in magenta.

To investigate this, we examined MS spectra of individual tryptic peptides from the different samples; for simplicity, we first describe spectra of peptides containing lysine but not arginine residues (Fig 1; see S1 and S2 Figs for corresponding MS/MS fragmentation spectra). For these peptides we observed a striking difference in MS spectra from cells grown in Arg-10 vs. Arg-0 or Arg-6. In the Arg-10 sample, peptide isotopic clusters contained several unexpected higher-molecular-mass peaks relative to the monoisotopic peak, such that the breadth of entire isotopic cluster was much greater than that of the equivalent isotopic envelopes seen in Arg-0 and Arg-6 samples (Fig 1A and 1B; compare panels i and ii vs. iii). Similar higher-molecular-mass peaks were also seen when cells were grown in medium containing Arg-10 and Lys-0, but not in medium containing Arg-0 and Lys-8 (data not shown). Based on these results, we conclude that the use of Arg-10, but not of Lys-8, increases the complexity of MS spectra, and that “heavy”-isotope label (i.e. label derived from Arg-10) is present in other amino acids. In other words, significant “conversion” of Arg-10 must be occurring. As mentioned above, and consistent with our previous work [2], such broadened isotopic clusters were not observed in samples grown in Arg-6 (Fig 1ii), suggesting that the proposed conversion may specifically involve ^15^N-labeled atoms. We also note that in these experiments, we used a concentration of arginine (30 mg/L) that is limiting for growth; thus, decreasing the arginine concentration would not be expected to ameliorate the problem.

**Fig 2:**
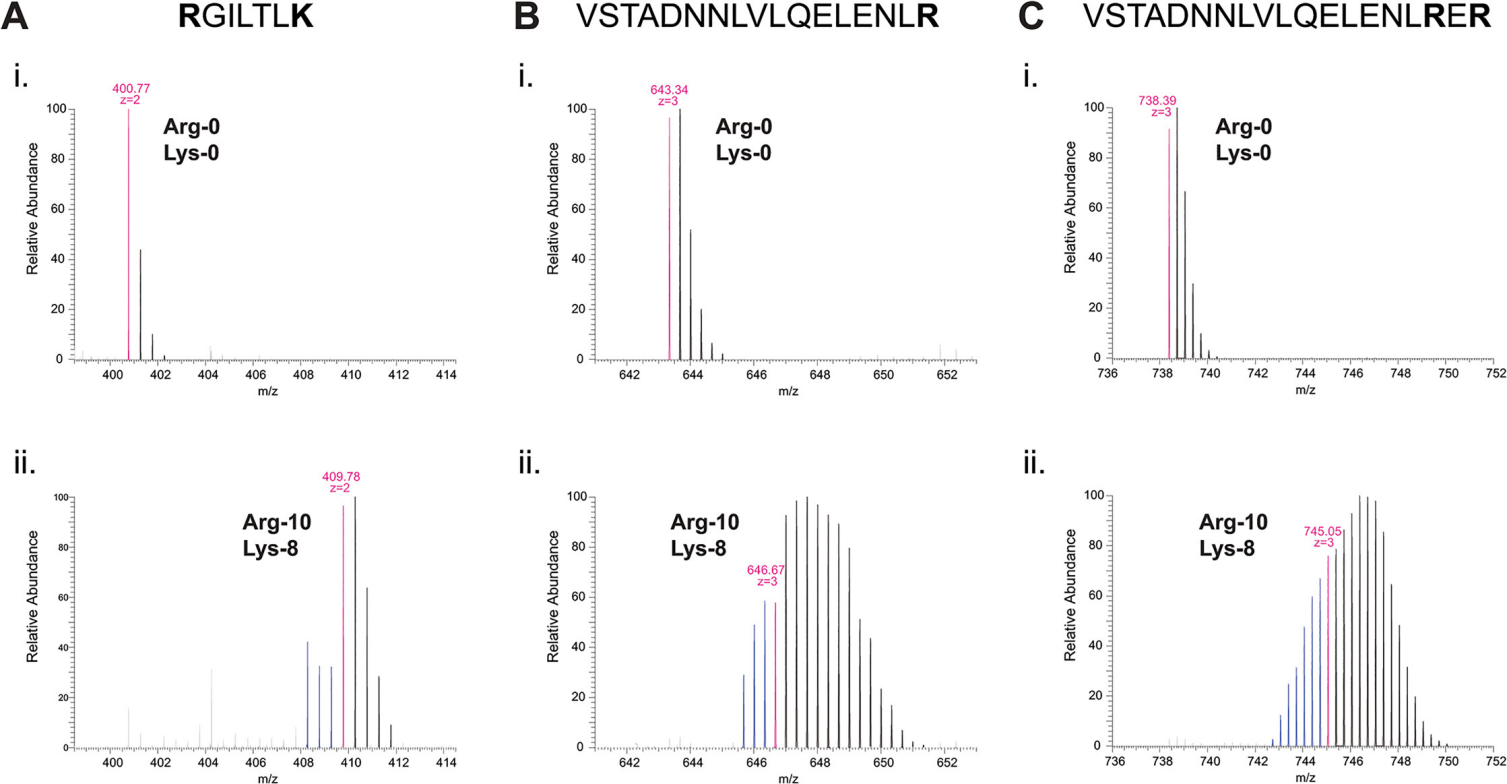
Arginine-containing peptides from cells grown in ^13^C_6_^15^N_4_-arginine (Arg-10) exhibit lower-molecular-mass peaks that suggest partial loss of “heavy” atoms from a portion of the Arg-10 pool. Mass spectra of tryptic peptides (RGILTLK, VSTADNNLVLQELENLR, and VSTADNNLVLQELENLRER in (A), (B), and (C), respectively) from *S*. *pombe* (A) actin and (B, C) Mto2[17A]-GFP, isolated from *car2∆ arg1-230 lys3-37* cells grown in either (i) unlabeled arginine (Arg-0) and unlabeled lysine (Lys-0), or (ii) ^13^C_6_^15^N_4_-arginine (Arg-10) and ^13^C_6_^15^N_2_-lysine (Lys-8), as indicated. In peptides from cells grown in Arg-10, several “pre-peaks” are observed (indicated in blue), with molecular masses significantly lower than the expected monoisotopic masses for Arg-10-labeled peptides. The number of pre-peaks is proportional to the number of arginine residues in the peptide (compare (B) and (C)). Mass-to-charge (m/z) ratios of monoisotopic peaks for each growth condition (i.e., assuming no conversion of labeled arginine) and inferred peptide charge-states are indicated in magenta.

When we examined MS spectra of peptides containing arginine residues (i.e. with or without lysine; Fig 2; see S3, S4 and S5 Figs for corresponding MS/MS fragmentation spectra), we found that in the Arg-10 sample, peptide isotopic clusters contained not only higher-molecular-mass peaks of the same type as mentioned previously, but also additional lower-molecular-mass peaks, corresponding to isotopologues up to 3 Da lighter than the expected monoisotopic masses for Arg-10-labeled peptides (Fig 2Aii and 2Bii). The appearance of these peaks, which we will refer to as “pre-peaks”, was even more pronounced in peptides containing two Arg-10 residues; in this case, isotopic clusters contained pre-peaks up to 6 Da lower than the monoisotopic mass (Fig 2Cii). As a consequence, monoisotopic peaks for peptides from Arg-10 samples were effectively “buried” within broad isotopic clusters (Fig 2ii). This would be expected to reduce success in identification of monoisotopic peaks for fragmentation, leading to an overall decrease in MS sensitivity.

**Fig 3:**
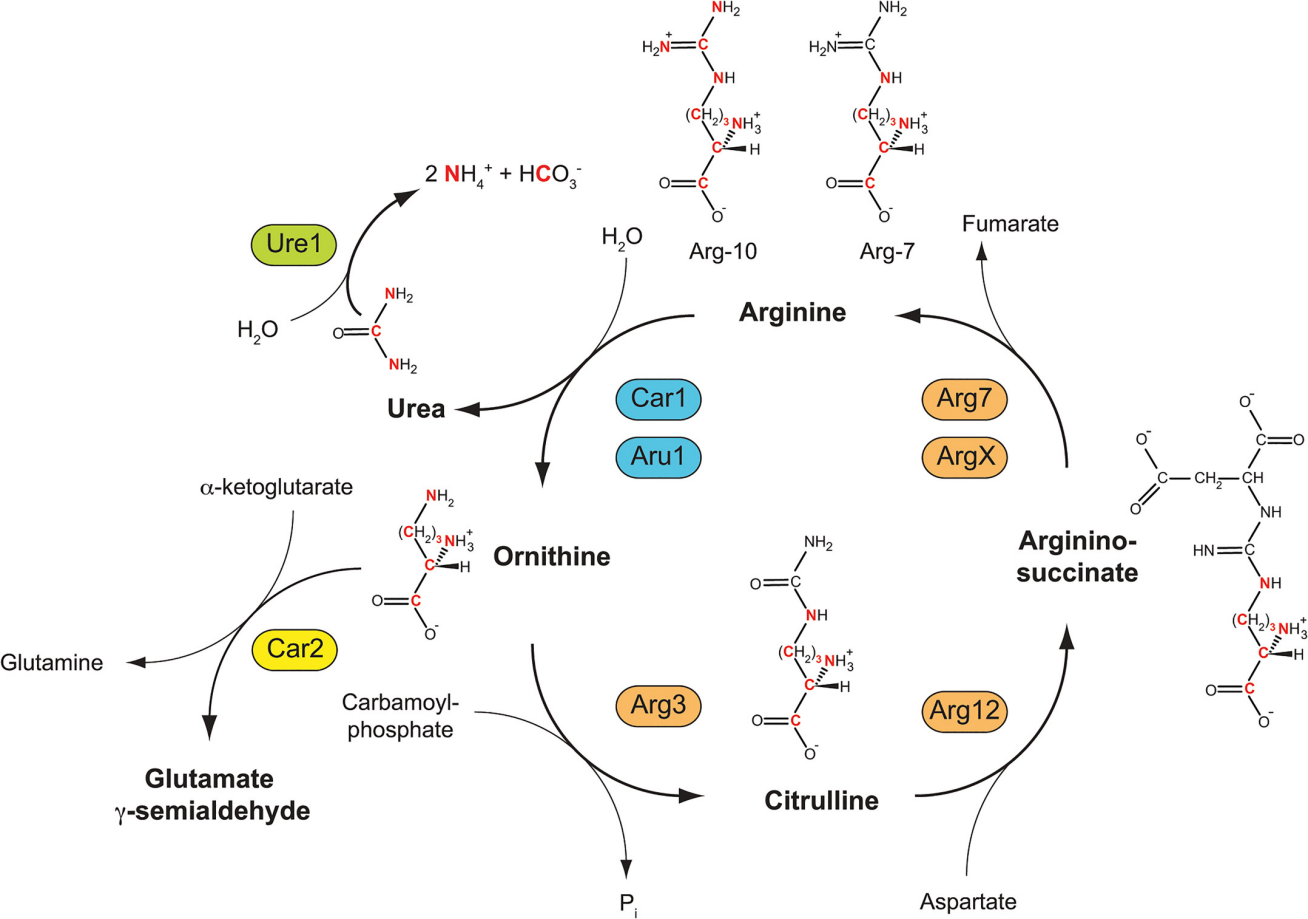
Anabolic and catabolic reactions from ^13^C_6_^15^N_4_-arginine (Arg-10) leading to formation of “heavy” ammonium and “heavy” bicarbonate, and resynthesis of ^13^C_5_^15^N_2_-arginine (Arg-7). Two S. pombe arginases, Car1 and Aru1, catalyze the conversion of Arg-10 into ^13^C_5_^15^N_2_-ornithine (“heavy” ornithine; “heavy” atoms are shown in red). This also produces “heavy” urea, which can be hydrolyzed by urease Ure1 to produce “heavy” ammonia and “heavy” carbon dioxide (shown here as ammonium and bicarbonate ions, respectively), which can subsequently be incorporated into additional amino acids. Ornithine can be converted back into arginine through citrulline and arginino-succinate intermediates, ultimately leading to formation of Arg-7.

Because peptides containing a single arginine residue displayed masses up to 3 Da lower than the expected monoisotopic mass of an Arg-10-containing peptide, while peptides containing two arginine residues displayed masses up to 6 Da lower, we hypothesized that *in vivo* in fission yeast, a portion of Arg-10 molecules may lose three out of their ten “heavy”-labeled atoms while retaining the remaining seven. Based on characterized metabolic pathways in the related fungi *Neurospora crassa* and *Saccharomyces cerevisiae* [24], such a loss of three “heavy” atoms could be due to activity of arginases, which hydrolyze arginine at the guanidinium group to produce ornithine and urea (Fig 3) [2]. In Arg-10-labeled cells, ornithine generated by arginase-dependent hydrolysis of Arg-10 would contain seven “heavy” atoms (^13^C_5_^15^N_2_-ornithine; Fig 3). Because ^13^C_5_^15^N_2_-ornithine can be converted back into arginine via citrulline and arginino-succinate intermediates, this would ultimately give rise to ^13^C_5_^15^N_2_-arginine (i.e. “Arg-7”; Fig 3). When incorporated into newly synthesized proteins, Arg-7 would produce peptide isotopologues 3 Da lighter than the “Arg-10 monoisotopic mass” when the peptide contains a single arginine residue, and 6 Da lighter when the peptide contains two arginine residues. This would provide a simple yet precise explanation for the pre-peaks observed in arginine-containing peptides from cells grown in Arg-10.

Similarly, the presence of higher-molecular-mass peaks in peptide isotopic clusters in Arg-10 samples can be explained by the metabolism of the other product of arginase activity, namely urea. Urea is hydrolyzed by urease to produce carbon dioxide (mainly as bicarbonate ion) and ammonium ion. Accordingly, urea generated by arginase-dependent hydrolysis of Arg-10 would contain three “heavy” atoms (^13^C_1_^15^N_2_-urea), which would then appear in the form of ^13^C_1_-bicarbonate and ^15^N_1_-ammonium (Fig 3). In *S*. *cerevisiae*, pyruvate carboxylases Pyc1 and Pyc2 catalyse carboxylation of pyruvate to oxaloacetate [25], a precursor of aspartate as well as asparagine, methionine, lysine and threonine [26]. A comparable process is likely to occur in *S*. *pombe*, via the uncharacterised pyruvate carboxylase Pyr1 (SPBC17G9.11c); thus, in *car2∆ arg1-230 lys3-37* cells grown in Arg-10, this would ultimately give rise to ^13^C_1_-amino acids. In addition, in wild-type (i.e. prototrophic) *S*. *pombe*, both urea and ammonium can serve as the sole source of nitrogen [27–29]. This implies that “heavy” nitrogen atoms from labeled ammonium should be incorporated into virtually all amino acids. Collectively, the incorporation of small-metabolite “heavy” atoms into amino acid pools would be expected to produce isotopologues with a broad, heterogeneous range of higher-than-expected molecular masses, as observed in all peptides from Arg-10 samples (Fig 1iii, Fig 2ii).

**Fig 4:**
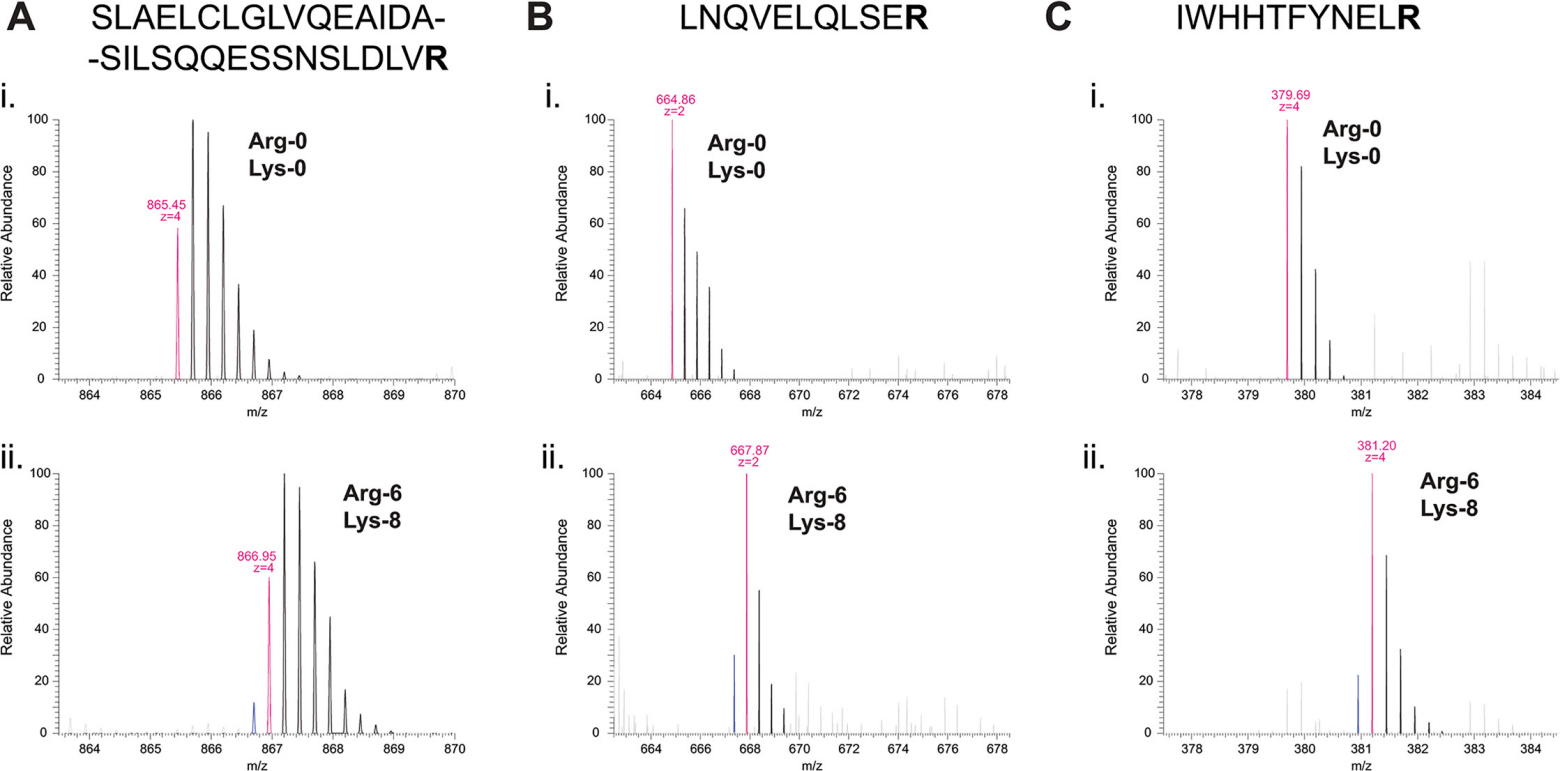
Arginine-containing peptides from cells grown in ^13^C_6_-arginine (Arg-6) exhibit a single lower-molecular-mass peak that indicates loss of a single “heavy” atom from a portion of the Arg-6 pool. Mass spectra of tryptic peptides (SLAELCLGLVQEAIDASILSQQESSNSLDLVR, LNQVELQLSER, and IWHHTFYNELR, in (A), (B) and (C), respectively) from *S*. *pombe* (A, B) Mto2[17A]-GFP and (C) actin, isolated from *car2∆ arg1-230 lys3-37* cells grown in either (i) unlabeled arginine (Arg-0) and unlabeled lysine (Lys-0), or (ii) ^13^C_6_-arginine (Arg-6) and ^13^C_6_^15^N_2_-lysine (Lys-8), as indicated. In peptides from cells grown in Arg-6, the single “pre-peak” (indicated in blue) indicates conversion of a portion of Arg-6 to ^13^C_5_-arginine (Arg-5). Masses of monoisotopic peaks for each growth condition (i.e., assuming no conversion of labeled arginine) and inferred peptide charge-states are indicated in magenta.

The mechanism that we propose here implies that in *car2∆ arg1-230 lys3-37* cells labeled with Arg-6, a similar arginine conversion should be observed; in this case, however, one would expect a portion of the Arg-6 pool to be converted to Arg-5, because the amino(imino)methyl group from Arg-6 contains only a single “heavy” ^13^C carbon atom (by contrast, the same group from Arg-10 contains one ^13^C carbon atom and two ^15^N nitrogen atoms). In the MS spectrum of a peptide containing an arginine residue, this should therefore result in the presence of a “pre-peak” with a molecular mass exactly 1 Da lower than the expected monoisotopic mass for an Arg-6-containing peptide (i.e., a “minus 1 pre-peak”). Interestingly, in MS spectra of arginine-containing peptides from Arg-6 samples, we observed a minus 1 pre-peak of precisely this nature (Fig 4; see S6, S7 and S8 Figs for corresponding MS/MS fragmentation spectra). Indeed, in our previous report [2], comparable minus 1 pre-peaks were also observed (see Fig 4E in [2]); however, at that time, these were attributed to impurities in commercially available “heavy” amino acids. In this context it is worth noting that Arg-6 conversion to Arg-5 does not actually have as significant an impact on peptide identification or quantification as does Arg-10 conversion to Arg-7. This is because isotopic envelopes of peptides labeled with Arg-6 and Arg-5 almost completely overlap (Fig 4), whereas this is clearly not the case for Arg-10 and Arg-7 (see Fig 2Aii, 2Bii and 2Cii).

**Fig 5:**
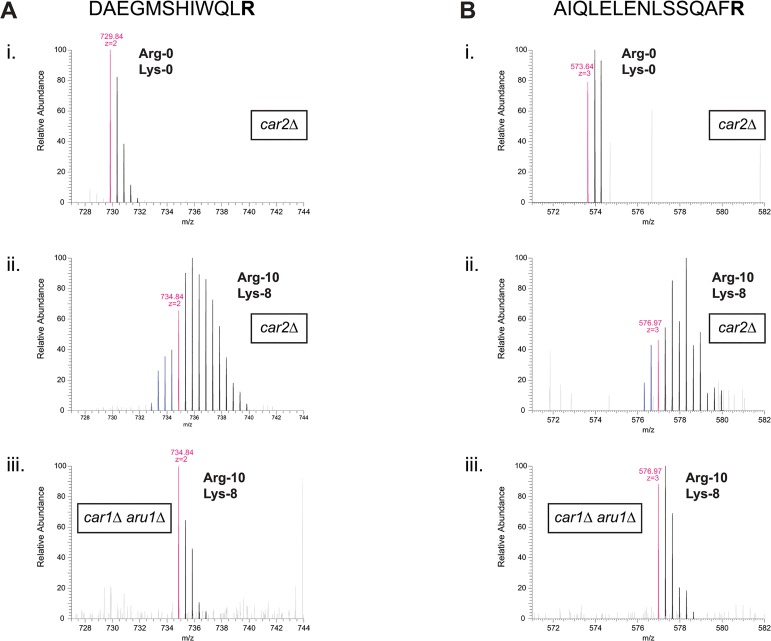
Arg-10 conversion is prevented in arginase-deficient cells. Mass spectra of tryptic peptides (DAEGMSHIWQLR and AIQLELENLSSQAFR in (A) and (B), respectively) from *S*. *pombe* Mto1 protein (SPCC417.07c) isolated from (i, ii) *car2∆ arg1-230 lys3-37* or (iii) *car1∆ aru1∆ arg1-230 lys3-37* cells, grown in either (i) unlabeled arginine (Arg-0) and unlabeled lysine (Lys-0), or (ii, iii) ^13^C_6_^15^N_4_-arginine (Arg-10) and ^13^C_6_^15^N_2_-lysine (Lys-8), as indicated. In peptides isolated from *car2∆* cells grown in Arg-10, extensive conversion is observed, resulting in both higher-molecular-mass peaks and lower-molecular-mass “pre-peaks” (ii; see also Figs 1 and 2). These are not observed in peptides from *car1∆ aru1∆* cells grown in Arg-10 (iii). Mass-to-charge (m/z) ratios of monoisotopic peaks for each growth condition (i.e., assuming no conversion of labeled arginine) and inferred peptide charge-states are indicated in magenta. Pre-peaks are indicated in blue.

In our original development of SILAC in fission yeast [2], we showed that conversion of Arg-6 to other amino acids could be prevented either by deletion of ornithine transaminase *car2+* or by double-deletion of arginases *car1+* and *aru1+*. At that time, use of the *car2∆* mutant was preferred primarily for reasons of simplicity (i.e. only one gene-deletion is required, instead of two). However, given the results described above, and our proposed mechanism to explain them, we reasoned that using a *car1∆ aru1∆* double mutant instead of the *car2∆* single mutant might solve the problems associated with Arg-10 labeling (see Fig 3). We therefore compared peptides after Arg-10 labeling in *car2∆ arg1-230 lys3-37* cells vs. *car1∆ aru1∆ arg1-230 lys3-37* cells. Strikingly, and in agreement with our hypothesis, in *car1∆ aru1∆ arg1-230 lys3-37* cells, conversion of Arg-10 was completely prevented; that is, we observed neither higher-molecular-mass peaks, attributed to ^15^N and ^13^C incorporation into additional amino acids via bicarbonate and/or ammonium, nor the “minus 3 pre-peaks”, attributed to Arg-10 conversion to Arg-7 (Fig 5; see S9 and S10 Figs for corresponding MS/MS fragmentation spectra). This was the case not only when *arg1-230* was used for arginine auxotrophy, but also when *arg3-D4* was used (data not shown). Thus, by deleting genes encoding arginases rather than the gene encoding ornithine transaminase, the problems associated with Arg-10 can be overcome.

In summary, here we have shown that the use of *car1∆ aru1∆* double mutants allows Arg-10 to be used for “heavy”-isotope labeling of fission yeast. As large-scale proteomics studies are becoming increasingly common in fission yeast, having tools to enable the best use of SILAC is of considerable importance. During preparation of this manuscript, an alternative method allowing use of Arg-10 in fission yeast was described by Carpy *et al*., in the context of triple-label SILAC [30]. In triple-label SILAC, a “light” (Arg-0, Lys-0) sample is mixed with a “medium” sample grown in D_4_-lysine (deuterated lysine; Lys-4) and Arg-6, and a “heavy” sample grown in Lys-8 and Arg-10. Because there is only a 4 Da mass difference between Lys-4 and Lys-8 as well as between Arg-6 and Arg-10, isotopic envelopes of peptides grown in the “medium” and “heavy” conditions are very close to each other. Therefore, for efficient analysis and quantification of triple-SILAC data, no conversion of either arginine or lysine should occur, in order to avoid overlap of the isotopic envelopes originating from these two different labeling states (which normally represent two completely different biological samples).

In their method, Carpy *et al*. combined *car2∆* [2] with deletion of the *nic1+* gene, which encodes a Ni^2+^ transporter (Nic1) required for full activity of Ni^2+^-dependent urease Ure1 (see Fig 3) under physiological conditions [31]. However, use of *nic1∆* cells for Arg-10 labeling did not completely eliminate the appearance of higher-molecular-mass peaks, presumably because low levels of Arg-10 were still being converted into ^15^N-ammonium [30]. Therefore, Carpy *et al*. also modified the nitrogen source of their SILAC media, replacing sodium glutamate as sole nitrogen source with a combination of sodium glutamate and (unlabeled) ammonium chloride, under the presumption that this unlabeled ammonium could outcompete ^15^N_1_-ammonium during amino acid anabolism. Use of this unconventional combination of nitrogen sources could be viewed as a disadvantage for physiological studies, but at the same time it should be acknowledged that in our own labeling method, although we use only ammonium as nitrogen source, it is used at a much lower concentration (6–9 mM) than is used in conventional *S*. *pombe* minimal medium (96 mM). Our change in medium formulation was introduced because high ammonium concentrations inhibit arginine uptake, while low ammonium concentrations are in fact sufficient for robust growth [2]. It is also not completely clear whether use of a *nic1∆* mutant may alter cell physiology or metabolism in unforeseen ways [30, 31], but the same criticism could in principle be applied to any approach involving genetic mutation. Finally, we note that in their work, Carpy *et al*. used an *arg3-D4* arginine auxotrophic mutant. Although they do not mention this in their paper, it is likely that in the context of their method, use of *arg1-230*, another commonly used arginine-auxotrophic mutation, would not have prevented Arg-10 conversion (i.e., to Arg-7) to the same extent. The reason for this is that ornithine carbamoyl transferase Arg3 is part of the “arginine re-synthesis pathway” (see Fig 3), and thus the *arg3-D4* mutation effectively prevents the formation of ^13^C_5_^15^N_2_-citrulline from ^13^C_5_^15^N_2_-ornithine. By contrast, use of *arg1-*230 would allow formation of ^13^C_5_^15^N_2_-citrulline, and thus “re-synthesis” of Arg-7.

Overall, our method is complementary to the one proposed by Carpy *et al*. It addresses the problem of conversion of both Arg-6 and Arg-10, and based on this we would argue that the *car1∆ aru1∆* genetic background should be preferred over *car2*∆ not only for Arg-10 labeling but also for Arg-6 labeling. Moreover, our method prevents not only the metabolism of ^15^N-labeled ammonium, but also the formation of labeled urea itself. The choice of which of these two labeling methods to use may depend on the exact experimental details involved, as well as the desired growth conditions. These issues highlight the fact that regardless of what specific approach is taken for “heavy”-isotope labeling, it is important to appreciate why a given approach works, and how various anabolic and catabolic processes may contribute to, or counteract, its success.

## Materials and Methods

### Yeast strain growth

*Schizosaccharomyces pombe* methods were as described [32]. “Heavy” isotope-labeled amino acids were: L-^13^C_6_^15^N_2_-lysine (Lys-8), L-^13^C_6_-arginine (Arg-6) and L-^13^C_6_^15^N_4_-arginine (Arg-10) (Sigma Isotec). Cells were grown in SILAC medium (EMM2 using 6 mM NH_4_Cl as nitrogen source, supplemented with 40 mg/L L-arginine (either Arg-0, Arg-6, or Arg-10, as indicated) and 30 mg/L L-lysine (either Lys-0 or Lys-8, as indicated)) [2]. Cells were harvested at OD_595_ = 2, which on our spectrophotometer corresponds to ~2.5x10^7^ cells/mL (late log phase).

To ensure all proteins are isotopically labeled, a small “heavy” pre-culture was used to inoculate the “heavy” culture, and the total number of generations grown in “heavy” media was typically more than 10. MS analysis was performed on immunoprecipitated samples.

### Immunoprecipitation

Fission yeast soluble extracts were made by freezing cell pellets in liquid nitrogen followed by grinding to a fine powder while frozen. Lysis buffer (25 mM sodium phosphate pH 7.5, 100 mM KCl, 0.5 mM EDTA, 0.2% Triton X-100, protease inhibitor cocktail (10 μg/mL of each: chymostatin, leupeptin, antipain, pepstatin, E64, 2 mM AEBSF, 2 mM benzamidine, 2 mM PMSF), and phosphatase inhibitors (50 mM Na β-glycerophosphate, 1mM NaF, 0.1 mM Na_3_VO_4_, 50 nM calyculin A, 50 nM okadaic acid) was then added to the cell powder and the cell powder was kept on ice until fully resuspended. Lysates were cleared by 2x15 minute centrifugation at 4000 rpm, and the total protein concentration was determined by Bradford assay. 1–10 mL of extract was used for immunoprecipitation. 3x10^7^ Protein G Dynabeads, previously covalently coupled with dimethyl pimelimidate to 1.2 μg of homemade sheep anti-GFP antibody was added per each 1 mL of extract. Beads were incubated with the lysate for 90 minutes, and then washed 6 times with 1 mL of the lysis buffer. Protein was eluted from beads by 15 min incubation at 50°C in Laemmli sample buffer, run on 10% SDS-PAGE gel and stained with Coomassie Blue.

### Sample processing for MS analysis

A protein band of Mto2[17A]-GFP was excised from a Coomassie Blue–stained gel. The protein was reduced, alkylated and digested with trypsin at an enzyme-to-protein ratio of 1:50, as described [33]. Peptides obtained during trypsin digestion were desalted using C18 StageTips [34, 35].

C18 material (ReproSil-Pur C18-AQ 3 μM; Dr Maisch GmbH, Ammerbuch-Entringen, Germany) was packed into an analytical column with a spray emitter (75-μm inner diameter, 8-μm opening, 250-mm length; New Objectives) using an air pressure pump (Proxeon Biosystems). Mobile phase A consisted of water and 0.1% formic acid and mobile phase B consisted of 80% ACN and 0.1% formic acid. Peptides were loaded onto the column with 2% B at 500 nL/min flow rate. Elution was performed at 300 nL/min flow rate with two gradients: linear increase from 2% B to 40% B in 79 minutes; then increase from 40% to 90% B in 11 minutes.

The eluted peptides were analysed either by Q-Exactive mass spectrometer (*car2∆*) or by Orbitrap Velos (*car1∆ aru1∆*).

Full MS Scans were acquired on the Q-Exactive mass analyser over the range m/z 300–1750 with a mass resolution of 70 000 (at m/z 200), with target value of 1.0E+06. From each MS survey scans, the ten most intense peaks with charge state ≥2 were fragmented in the HCD collision cell with normalized collision energy of 25%, and MS/MS scans were acquired with a mass resolution of 35,000 at m/z 200 and target value of 5.0E+05. The ion selection threshold was 2.1E+04 counts, and the maximum allowed ion accumulation times were 20 ms for full MS scans and 120 ms for FT MS/MS spectra. The dynamic exclusion time: 45 seconds, repeat count equal to 1.

The data acquisition was performed in a data-dependent manner over the range m/z 300–1800 on the Orbitrap Velos. The ten most intense precursor ions with charge state ≥2 were selected for fragmentation. MS and MS/MS scans were acquired in an Orbitrap mass analyser, and the peptides were fragmented by HCD with normalized collision energy of 40%. MS scans were acquired at a resolution of 100,000 at 400 m/z, while MS/MS spectra were acquired with a mass resolution of 7500. The automatic gain control for full FT MS was set to 5.0E+05 ions and for FT MS/MS was set to 1.0E+05 ions. The maximum allowed time for ion accumulation were 500 ms and 200 ms, respectively.

The generated peak lists were searched against protein databases using Mascot 2.0. XiSPEC Spectrum Viewer (http://spectrumviewer.org/) was used to visualize MS/MS spectra for figure presentation. Due to XiSPEC software design, occasionally b or y ions that are indicated in the peptide sequence (and shown as red peaks in MS/MS spectra) are not annotated; this is most likely to occur when peaks are very low intensity (especially if multiply charged) and/or very close to neighboring peaks.

## Supporting Information

S1 Fig Fragmentation spectra of peptides shown in Fig 1A. MS/MS spectra of a tryptic peptide (TIFFKDDGNYK) from *S*. *pombe* Mto2[17A]-GFP fusion protein isolated from *car2∆ arg1-230 lys3-37* cells grown in either unlabeled arginine (Arg-0) and unlabeled lysine (Lys-0), ^13^C_6_-arginine (Arg-6) and ^13^C_6_^15^N_2_-lysine (Lys-8), or ^13^C_6_^15^N_4_-arginine (Arg-10) and ^13^C_6_^15^N_2_-lysine (Lys-8), as indicated.
(EPS)

S2 Fig Fragmentation spectra of peptides shown in Fig 1B. MS/MS spectra of a tryptic peptide (GIDFKEDGNILGHK) from *S*. *pombe* Mto2[17A]-GFP fusion protein isolated from *car2∆ arg1-230 lys3-37* cells grown in either unlabeled arginine (Arg-0) and unlabeled lysine (Lys-0), ^13^C_6_-arginine (Arg-6) and ^13^C_6_^15^N_2_-lysine (Lys-8), or ^13^C_6_^15^N_4_-arginine (Arg-10) and ^13^C_6_^15^N_2_-lysine (Lys-8), as indicated.
(EPS)

S3 Fig Fragmentation spectra of peptides shown in Fig 2A. MS/MS spectra of a tryptic peptide (RGILTLK) from *S*. *pombe* actin, isolated from *car2∆ arg1-230 lys3-37* cells grown in either unlabeled arginine (Arg-0) and unlabeled lysine (Lys-0), or ^13^C_6_^15^N_4_-arginine (Arg-10) and ^13^C_6_^15^N_2_-lysine (Lys-8), as indicated.
(EPS)

S4 FIg Fragmentation spectra of peptides shown in Fig 2B. MS/MS spectra of a tryptic peptide (VSTADNNLVLQELENLR) from *S*. *pombe* Mto2[17A]-GFP, isolated from *car2∆ arg1-230 lys3-37* cells grown in either unlabeled arginine (Arg-0) and unlabeled lysine (Lys-0), or ^13^C_6_^15^N_4_-arginine (Arg-10) and ^13^C_6_^15^N_2_-lysine (Lys-8), as indicated.
(EPS)

S5 Fig Fragmentation spectra of peptides shown in Fig 2C. MS/MS spectra of a tryptic peptide (VSTADNNLVLQELENLRER) from *S*. *pombe* Mto2[17A]-GFP, isolated from *car2∆ arg1-230 lys3-37* cells grown in either unlabeled arginine (Arg-0) and unlabeled lysine (Lys-0), or ^13^C_6_^15^N_4_-arginine (Arg-10) and ^13^C_6_^15^N_2_-lysine (Lys-8), as indicated.
(EPS)

S6 Fig Fragmentation spectra of peptides shown in Fig 4A. MS/MS spectra of a tryptic peptide (SLAELCLGLVQEAIDASILSQQESSNSLDLVR) from *S*. *pombe* Mto2[17A]-GFP, isolated from *car2∆ arg1-230 lys3-37* cells grown in either unlabeled arginine (Arg-0) and unlabeled lysine (Lys-0), or ^13^C_6_-arginine (Arg-6) and ^13^C_6_^15^N_2_-lysine (Lys-8), as indicated.
(EPS)

S7 Fig Fragmentation spectra of peptides shown in Fig 4B. MS/MS spectra of a tryptic peptide (LNQVELQLSER) from *S*. *pombe* Mto2[17A]-GFP, isolated from *car2∆ arg1-230 lys3-37* cells grown in either unlabeled arginine (Arg-0) and unlabeled lysine (Lys-0), or ^13^C_6_-arginine (Arg-6) and ^13^C_6_^15^N_2_-lysine (Lys-8), as indicated.
(EPS)

S8 Fig Fragmentation spectra of peptides shown in Fig 4C. MS/MS spectra of a tryptic peptide (IWHHTFYNELR) from *S*. *pombe* actin, isolated from *car2∆ arg1-230 lys3-37* cells grown in either unlabeled arginine (Arg-0) and unlabeled lysine (Lys-0), or ^13^C_6_-arginine (Arg-6) and ^13^C_6_^15^N_2_-lysine (Lys-8), as indicated.
(EPS)

S9 Fig Fragmentation spectra of peptides shown in Fig 5A. MS/MS spectra of a tryptic peptide (DAEGMSHIWQLR) from *S*. *pombe* Mto1 protein (SPCC417.07c) isolated from *car2∆ arg1-230 lys3-37* or *car1∆ aru1∆ arg1-230 lys3-37* cells, grown in either unlabeled arginine (Arg-0) and unlabeled lysine (Lys-0), or ^13^C_6_^15^N_4_-arginine (Arg-10) and ^13^C_6_^15^N_2_-lysine (Lys-8), as indicated.
(EPS)

S10 Fig Fragmentation spectra of peptides shown in Fig 5B. MS/MS spectra of a tryptic peptide (AIQLELENLSSQAFR) from *S*. *pombe* Mto1 protein (SPCC417.07c) isolated from *car2∆ arg1-230 lys3-37* or *car1∆ aru1∆ arg1-230 lys3-37* cells, grown in either unlabeled arginine (Arg-0) and unlabeled lysine (Lys-0), or ^13^C_6_^15^N_4_-arginine (Arg-10) and ^13^C_6_^15^N_2_-lysine (Lys-8), as indicated.
(EPS)
